# Feed-Forward Neural Network Soft-Sensor Modeling of Flotation Process Based on Particle Swarm Optimization and Gravitational Search Algorithm

**DOI:** 10.1155/2015/147843

**Published:** 2015-10-25

**Authors:** Jie-Sheng Wang, Shuang Han

**Affiliations:** School of Electronic and Information Engineering, University of Science and Technology Liaoning, Anshan, Liaoning 114044, China

## Abstract

For predicting the key technology indicators (concentrate grade and tailings recovery rate) of flotation process, a feed-forward neural network (FNN) based soft-sensor model optimized by the hybrid algorithm combining particle swarm optimization (PSO) algorithm and gravitational search algorithm (GSA) is proposed. Although GSA has better optimization capability, it has slow convergence velocity and is easy to fall into local optimum. So in this paper, the velocity vector and position vector of GSA are adjusted by PSO algorithm in order to improve its convergence speed and prediction accuracy. Finally, the proposed hybrid algorithm is adopted to optimize the parameters of FNN soft-sensor model. Simulation results show that the model has better generalization and prediction accuracy for the concentrate grade and tailings recovery rate to meet the online soft-sensor requirements of the real-time control in the flotation process.

## 1. Introduction

Flotation is known as froth flotation, and it is a physicochemical reaction process. Flotation is the process which is based on the differences of the surface property of solid materials to separate useful minerals and gangue by means of the buoyancy of air bubbles from ore pulp by this method to improve the concentrate grade [1]. In the production process of flotation, concentrate grade and other economic and technical indicators are key control indicators of the production process. Process control indicators of domestic flotation process are mainly based on an experienced operator to observe the information (such as foam color, size, flow rate, and texture features) which is provided by the bubble state formed on the surface of the flotation tank and to adjust the flotation level and change agents system [2, 3]. Inference estimate (soft-sensor) technology can effectively solve the online estimation problems where the flotation process is difficult to online measure the economic and technical indicators.

Domestic and foreign scholars carry through the research on soft-sensor modeling of the key technical indicators in the flotation process and make a lot of achievements [4–16]. Hargrave and Hall study the diagnosis and analysis methods of the metal grade, quality, and flow rate in flotation process by using the color and surface tissue [4]. Bartolacci et al. use multivariate image analysis (MIA) and partial least squares (PLS) methods to establish the experience prediction model of flotation grade [5]. Morar et al. utilize the machine vision method to predict the performance of the flotation process, such as concentrate grade and tailings recovery rate [6]. Moolman and many other scholars created a bubble dynamic model based on image processing through researching flotation foam structure and calculated the content of useful minerals in foam through this model [7].

At home, Yang et al. put forward a bubble image segmentation method based on the clustering presplit and the accuracy distance reconstruction [8]. In that the soft-sensor method with multiple models can improve the overall prediction accuracy and have the characteristic of robustness; Wang et al. present a multi-T-S fuzzy neural network soft-sensor model of flotation process based on the FCM clustering algorithm [9]. Yang et al. use the flotation froth video image features as auxiliary variables and establish a soft-sensor model of the flotation pulp pH value based on the sparse polynuclear least squares support vector machine (SVM) and use Schmidt orthogonalization theory to reduce the multinuclear matrix [10]. Li et al. set up a soft-sensor mode by combining the principal component analysis (PCA) and extreme learning machine (ELM) methods [11]. Zhou et al. extracted color and size characteristics of the foam by using digital image processing method and established a recovery prediction model [12]. Wang and Zhang proposed a kind of soft-sensor model of economic and technical index based on PCA and ANFIS and combining PSO algorithm with LSM put forward a new learning process to optimize parameters of ANFIS [13]. Geng and Chai utilized least squares support vector machine to establish soft-sensor model of concentrate grade and tailing grade in the flotation process based on analyzing related influencing factors of concentrate grade and tailing grade of the flotation process technology indicators [14]. Wang et al. proposed the features extraction method of flotation froth images and BP neural network soft-sensor model of concentrate grade optimized by shuffled cuckoo searching algorithm [15]. Wang et al. proposed an echo state network (ESN) based fusion soft-sensor model optimized by the improved glowworm swarm optimization (GSO) algorithm. Simulation results show that the model has better generalization and prediction accuracy [16].

This paper proposes a feed-forward neural network (FNN) soft-sensor model by using process datum in the flotation process for predicting the flotation concentrate grade and recovery rate, which is optimized by the PSO-GSA algorithm. Simulation results verify the validity of the proposed soft-sensor model. The paper is organized as follows. In Section 2, the technique flowchart of flotation process is introduced. The FNN soft-sensor model of flotation process optimized by PSO-GSA algorithm is presented in Section 3. In Section 4, experiment and simulation results are introduced in detail. Finally, the conclusion illustrates the last part.

## 2. Technique Flowchart of Flotation Process

**Figure 1:**
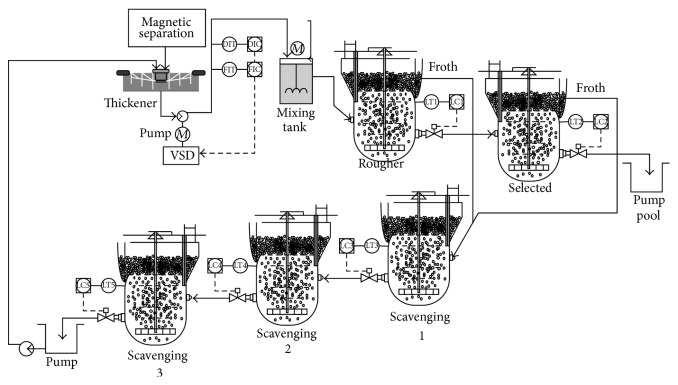
Technique flowchart of flotation process.

Flotation process is used to separate useful minerals and gangue based on the differences of the surface property of solid materials.  Figure 1 is a typical iron ore flotation process consisting of the roughing, concentration, and scavenging [11]. The system input is the fine concentrate pulp which is early output of beneficiation process in the forepart. The pulp density is about 38% and concentrate grade is about 64%. Inlet pulp is fed into the high-stirred tank through the pulp pipeline by feed pump. At the same time, the flotation reagent according to a certain concentration ratio is also fed into high-stirred tank through dosing pump. On the other hand, the pulp temperature must reach a suitable flotation temperature by heating. If the dosage is appropriate, the flotation cells can output a grade of 68.5%–69.5% concentrate [15, 16].

The control objective of flotation process is to ensure the concentrate grade and the tailings recovery rate are within a certain target range. In common, based on the offline artificial laboratory to get grade values, the operators adjust the flotation cell level and the amount of flotation reagent addition. Due to the artificial laboratory for two hours at a time, when the process variables and boundary conditions change in the flotation process, they cannot timely adjust the flotation operation variables, which results in such phenomena that the flotation concentrate grade and the tailings recovery rate are too high or too low [15, 16]. By analyzing the flotation technique, the process variables and boundary conditions mainly include feed grade *x*_1_, feed flow rate *x*_2_, feed concentration *x*_3_, feed granularity *x*_4_, and medicament flow rate *x*_5_. The modeling data is shown in Table 1.

## 3. Soft-Sensor Modeling of Flotation Process

### 3.1. Structure of Soft-Sensor Model

**Figure 2:**
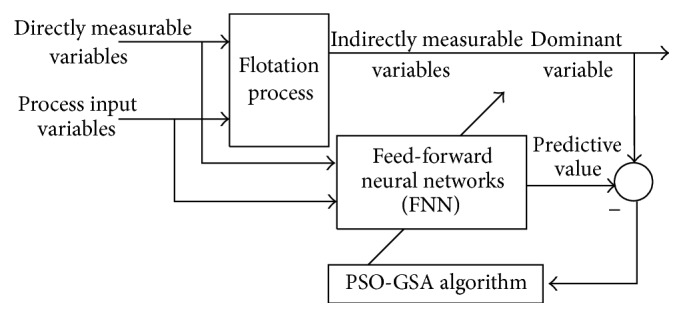
Structure of the proposed soft-sensor model of flotation process.

The structure of the proposed feed-forward neural network (FNN) soft-sensor model of the flotation process based on PSO-GSA algorithm is shown in Figure 2.

The auxiliary variables of soft-sensor model proposed in this paper are feed grade, feed concentration, feed flow, feed granularity, and medicament flow. Then the samples composed of the auxiliary variable are normalized as the model input. Finally, parameters of FNN soft-sensor model are optimized by PSO-GSA algorithm. Thereby, the accurate prediction of concentrate grade and tailing recovery rate of the flotation process is achieved. Considering a multi-input single-output (MISO) system, the training set can be represented as *D* = {*Y*, *X*_*i*_∣*i* = 1,2,…, *m*}, where *Y* represents the output and *X*_*i*_ denotes the *i*th input vector and it can be represented as *X*_*i*_ = [*x*_1*i*_, *x*_2*i*_,…,*x*_*ni*_]′ (*n* is the number of the training samples and *m* is the number of the input variables). The establishment of soft-sensor model needs a data set from the normal working condition as the modeling data. Assuming the *m* process variables, *n* data vector samples comprise the test data matrix *X* ∈ *R* ^*n*×*m*^. In order to avoid the effect of different dimensions of process variables for results and be convenient for mathematical handling, it is necessary to normalize the data. Assume the mean vector of *X* is *μ* and the standard deviation vector is *σ*. The process variables after the normalization are described as follows:

(1) $$\begin{array}{c} \hat{X}=\frac{\left(X-\mu \right)}{\sigma }. \end{array}$$

Then the input vectors $\hat{X}$ of the training samples are fed into FNN soft-sensor model to obtain the predictive output $\hat{Y}$. On the other hand, the root mean square error (RMSE) is adopted as the fitness value of soft-sensor model:

(2) $$\begin{array}{c} RMSE=\sqrt{\overset{n}{\underset{k=1}{\sum }}\frac{{\left({\hat{Y}}_{k}-Y_{k}^{*}\right)}^{2}}{n}}, \end{array}$$

where *Y* ^*∗*^ is the actual output of the training samples.

### 3.2. Feed-Forward Neural Network (FNN)

**Figure 3:**
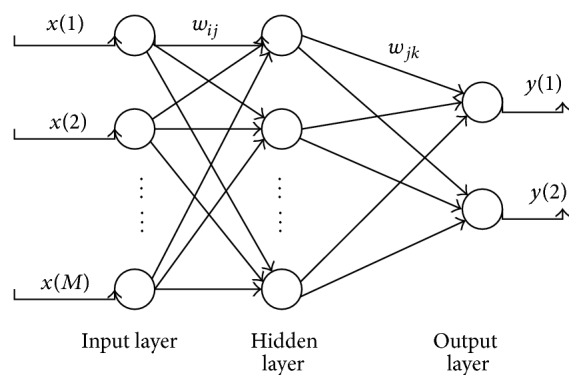
Structure of feed-forward neural network (FNN).

In accordance with different layers of the feed-forward neural network (FNN), it can be divided into single-layer feed-forward neural network and multilayer feed-forward neural network. The multilayer FNN is adopted in this paper, which includes an input layer, a hidden layer, and an output layer, whose structure is shown in Figure 3.

Assume that the input layer has *M* inputs; *i* (*i* = 1,2,…, *M*) represents any input. The hidden layer has *N* inputs; *j* (*j* = 1,2,…, *N*) represents any inputs. The connection weight values between the input layer and the hidden layer are *w*_*ij*_  (*i* = 1,2,…, *M*; *j* = 1,2,…, *N*). The connection weight values between the hidden layer and the output layer are *w*_*jk*_  (*k* = 1,2). Assume that the input of the hidden layer neurons is *u*_*j*_; output is *v*_*j*_. Input of output layer neurons is *o*_*k*_; total output is *y*_*k*_. So the calculation of FNN can be represented as follows:

(3) uj∑i=1Mwijxi,vjfuj=f∑i=1Mwijxi,ok∑j=1Nwjkvj,ykfok=f∑j=1Nwjkvj,

where *f*() represents the transfer function between input and output of the hidden layer and the output layer, which is also called the activation function.

### 3.3. FNN Soft-Sensor Model Optimized by Hybrid PSO-GSA Algorithm

#### 3.3.1. Particle Swarm Optimization (PSO) Algorithm

Particle swarm optimization (PSO) algorithm is a kind of swarm intelligent optimization algorithm, which is inspired by the birds' migration and swarming behavior during the foraging process. Due to its simplicity, it has been widely used in many optimization problems. It uses a large number of particles (potential solutions) to find the best solution in the search space, where each particle corresponds to a fitness value, and the velocity of the particles is decided by their flight direction and distance. Adjust the individual best value and the global best value to meet the requirements dynamically [17, 18].

Assume that, in an *S*-dimensional searching space, *n* particles consist of the population *X* = (*x*_1_, *x*_2_,…, *x*_*n*_), where the *i*th particle is expressed as an *S*-dimensional vector *x*_*i*_ = (*x*_*i*1_, *x*_*i*2_,…,*x*_*is*_)^*T*^. It represents the position of the *i*th particle in the searching space (the potential solutions of the discussed problem). *v*_*i*_ = (*v*_*i*1_, *v*_*i*2_,…,*v*_*is*_)^*T*^ represents the velocity of the *i*th particle, *pbest*_*i*_ = (*p*_*i*1_, *p*_*i*2_,…,*p*_*is*_)^*T*^ represents the individual best value, and *gbest* = (*g*_1_, *g*_2_,…,*g*_*s*_)^*T*^ represents the global best value. According to the following equations, the position and velocity vector of the particles are updated:

(4) vit+1=wvit+c1×rand×pbesti−xit+c2×rand×gbest−xit,

(5) xit+1=xit+vit+1,

where *w* is the inertia weight, *t* is the number of iterations, *v*_*i*_^*t*^ represents the velocity of *i*th particle in *t* iteration, *c*_1_ is the particle's acceleration weighting coefficient, *c*_2_ is a global acceleration weighting coefficient, *c*_1_ and *c*_2_ are learning factors (usually *c*_1_ = *c*_2_ = 2), rand is a random number between 0 and 1, and *x*_*i*_^*t*^ represents the current location of the *i*th particle in *t*th iteration.

The first part *wv* of (4) represents the search capability of PSO algorithm, and the second part *c*_1_ × rand × (*pbest*_*i*_ − *x*_*i*_^*t*^) and the third part *c*_2_ × rand × (*gbest* − *x*_*i*_^*t*^) represent individual and global optimization ability of particles. In the searching space, the position vector of the particles is randomly generated. In each iteration, (4) is used to update the velocity vector of particles. After determining the velocity of the particles, the particle position vector is updated by (5). The position vector of the particles will be constantly changed until the termination condition is satisfied.

#### 3.3.2. Gravitational Search Algorithm (GSA)

**Figure 4:**
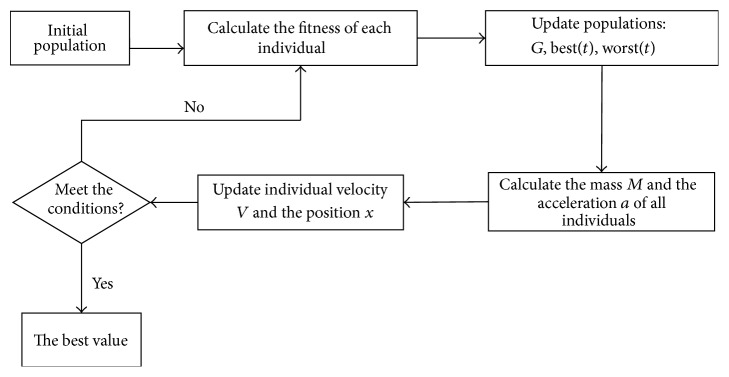
Flowchart of gravitation search algorithm.

In 2009, Rashedi et al. put forward a gravitational search algorithm (GSA), which is a heuristic optimization algorithm [19]. It uses the physics law to find the optimal solution in the searching space. Inspiration of the GSA comes from Newton's universal gravitation law. Gravity is a force of attraction that exists between any two masses, any two bodies, or any two particles. The size of the gravitational force is proportional to their product of the quality and inversely proportional to the distance between them. In this algorithm, each individual represents a potential solution, each of the potential solutions corresponds to a fitness value, and the fitness value is represented by the quality of the individual. Everything with massive particle in the universe attracts all other massive particles; a large mass of individuals is subject to greater gravitation. So a large mass of individuals is near to the global best value. The flowchart of gravitation search algorithm is described as in Figure 4.

Suppose, in an *n*-dimensional searching space, *N* substances constitute a population. Each individual's position (potential solution) is defined as follows:

(6) $$\begin{array}{c} x_{i}=\left(\;x_{i}^{1},\ldots ,x_{i}^{d},\ldots ,x_{i}^{n}\;\right),\;i=1,2,\ldots ,N, \end{array}$$

where *x*_*i*_^*d*^ is the position of substance *i* in *d*-dimension of the space.

In the searching space, all individuals are randomly placed in the *t*th generation. So the gravity of substance *j* attracting substance *i* in *d*-dimensional space is defined as

(7) $$\begin{array}{c} F_{ij}^{d}\left(\;t\;\right)=G\left(\;t\;\right)\frac{M_{pi}\left(t\right)\times M_{aj}\left(t\right)}{R_{ij}\left(t\right)+\varepsilon }\left(\;x_{j}^{d}\left(\;t\;\right)-x_{i}^{d}\left(\;t\;\right)\;\right), \end{array}$$

where *M*_*aj*_ represents the active gravitational mass of individual *j*, *M*_*pi*_ represents the passive gravitational mass of individual *i*, *G*(*t*) represents the gravitational constant in *t*th generation, *ε* is a small constant, and *R*_*ij*_(*t*) represents the Euclidean distance between substance *i* and substance *j*.

The gravitational constant *G* and the Euclidean distance between substance *i* and substance *j* are calculated as follows:

(8) Gt=G0×exp⁡−α×itermaxiter⁡,Rij=xit,xjt2,

where *α* is the decreasing coefficient (constant), *G*_0_ represents the initial gravitational constant, iter represents the number of current iterations, and maxiter represents the number of maximum iterations.

In the *d*-dimensional searching space, all gravity which acts on the material *i* is calculated as follows:

(9) $$\begin{array}{c} F_{i}^{d}\left(\;t\;\right)=\overset{N}{\underset{j=1,j\neq i}{\sum }}{rand}_{j}F_{ij}^{d}\left(\;t\;\right), \end{array}$$

where rand_*j*_ is a random number between 0 and 1.

According to Newton's motion law, the acceleration of the material *i* is proportional to force in *d* dimension and inversely proportional to the inverse of the mass. The acceleration of substance is calculated as follows:

(10) $$\begin{array}{c} a_{i}^{d}\left(\;t\;\right)=\frac{F_{i}^{d}\left(t\right)}{{M_{ii}\left(t\right)}^{\prime }}, \end{array}$$

where *t* represents the number of current iterations and *M*_*i*_ represents the mass of substance *i*. Speed and position of substance *i* are updated by the following equations:

(11) vidt+1=randi×vidt+aidt,

(12) xidt+1=xidt+vidt+1,

where rand_*i*_ is a random number between 0 and 1.

It can be seen, from the above two equations, that the current speed of a substance is defined as the part of the final speed (0 ≤ rand_*i*_ ≤ 1) and its acceleration. The current position of substance is equal to its final velocity and the current position. The fitness represents the quality of the material, which means that the greater the quality of the material, the higher the efficiency. According to the above formula, the heavier the material, the greater the gravity and the slower the movement. The quality of materials is updated by using the following equation:

(13) $$\begin{array}{c} m_{i}\left(\;t\;\right)=\frac{{fit}_{i}\left(t\right)-worst\left(t\right)}{best\left(t\right)-worst\left(t\right)}, \end{array}$$

where fit_*i*_(*t*) represents the fitness value of substance *i* in *t*th generation, best(*t*) represents the best individual fitness value in *t*th generation, and worst(*t*) represents the worst individual fitness value. With regard to the minimization problem, best(*t*)  and worst(*t*) are calculated as follows:

(14) bestt=minj∈1,…,N⁡fitjt,worstt=maxj∈1,…,N⁡fitjt.

The standardization of quality is defined by the following formula:

(15) $$\begin{array}{c} M_{i}\left(\;t\;\right)=\frac{m_{i}\left(t\right)}{\sum_{j=1}^{N}m_{j}\left(t\right)}. \end{array}$$

#### 3.3.3. PSO-GSA Algorithm

Although GSA has better optimization capability, the material appeared to have low convergence in the process of moving to the optimal value and to be easy to fall into local optimum phenomenon. So PSO algorithm is used to update the position and velocity of the individual in order to make up this shortcoming of GSA. The basic idea of PSO-GSA is described as follows. Firstly, generate the initial position vector *X* = (*x*_1_, *x*_2_,…, *x*_*n*_) and velocity vector *v*_*i*_ = (*v*_*i*1_, *v*_*i*2_,…,*v*_*is*_)^*T*^ of *N* individuals randomly. According to the initial positions of all individuals, calculate the fitness value corresponding to each individual and record the best fitness value best(*t*) and the worst fitness value worst(*t*) and the corresponding position vector of the individuals. The quality of individuals is calculated by (13)–(15). Then calculate the gravitational constant *G* and the Euclidean distance between two individuals and the individual's gravitation *F* and acceleration *a*. At this time, according to (11)-(12), the global search ability of PSO algorithm is used to update velocity and position of the individual, and then the fitness value and the corresponding optimal value are calculated. The best individual is obtained until the number of iterations is reached. Consider

(16) vit+1=w×vit+c1′×rand×acit+c2′×rand×gbest−xit,

where *v*_*i*_(*t*) represents the velocity of the material *i* in *t*th generation, *c*_*j*_′ is an acceleration coefficient, *w* is the inertia weight, rand represents a random number between 0 and 1, *ac*_*i*_(*t*) indicates the acceleration of substance *i* in *t*th generation, and *gbest* represents the optimal solution so far.

After updating the velocity vector, the location vector of substance is updated based on the following equation:

(17) $$\begin{array}{c} x_{i}\left(\;t+1\;\right)=x_{i}\left(\;t\;\right)+v_{i}\left(\;t+1\;\right). \end{array}$$

#### 3.3.4. Procedure of PSO-GSA Algorithm Optimizing FNN

**Figure 5:**
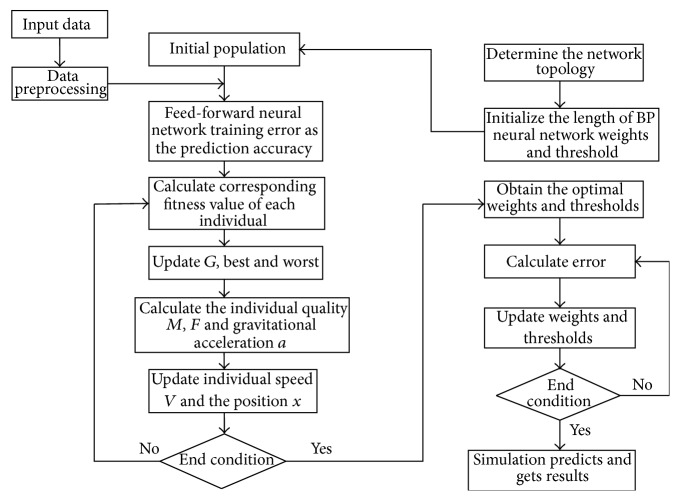
Flowchart of PSO-GSA optimizing FNN.

In this paper, the PSO-GSA hybrid algorithm is applied to optimize the parameters of the FNN soft-sensor model, whose aim is to improve the convergence speed and predictive accuracy. PSO-GSA algorithm is different from GSA. It adopts PSO algorithm to update its velocity and position, until it reaches the number of iterations or accuracy. Because the prediction accuracy of FNN soft-sensor model is related to the initial connection weights and thresholds, if the parameters are improper, it will lead to decline of prediction accuracy. Therefore, the hybrid PSO-GSA algorithm is used to optimize FNN soft-sensor model. The flowchart of PSO-GSA algorithm optimizing FNN is shown in Figure 5.

The algorithm procedure is described as follows in detail.

**Step 1 (initialize parameters).**

Determine the topology of FNN. Initialize the weights *w* and the threshold value *b* of FNN and predispose the training samples of flotation process. Initialize the population size *N* and the number of iterations *t*.

**Step 2 (generate the population randomly).**

Set the initial position *X* = (*x*_1_, *x*_2_,…, *x*_*n*_) and initial velocity *v*_*i*_ = (*v*_*i*1_, *v*_*i*2_,…,*v*_*is*_)^*T*^, the learning factors *c*_1_ and *c*_2_, and the inertia weight *w*. Initialize the global best value *gbest* and individual best value *pbest*, the decreasing coefficient *α*, and the gravitational constant *G*_0_.

**Step 3 .**

The position of each individual corresponds to a set of weights and thresholds of FNN soft-sensor model. Train FNN and calculate the fitness value fit(*t*), of each individual. Find the optimal fitness value best(*t*) and the worst fitness value worst(*t*) and record the best position *gbest*.

**Step 4 .**

According to (13)–(15), calculate the mass of the individual, the gravitational constant *G*, and the Euclidean distance between two individuals. Then calculate the gravitation *F* and the acceleration *a* of the individual. At this time, according to (11)-(12), the global search ability of PSO algorithm is adopted to update the velocity and position of the individuals. Then calculate the corresponding fitness value fit(*t*) and the optimal value *gbest*′ of the individual. The optimal value *gbest*′ is compared with the optimal value *gbest* in Step 3; the optimal individual after comparison is recorded as *gbest*′′.

**Step 5 .**

Determine whether the termination condition is reached or not (the objective function reaches a certain value or the number of iterations reaches the maximum). If the termination condition is not met, the procedure returns to Step 3.

**Step 6 (model validation).**

The corresponding parameters of the best individual *gbest* are set as the weights and thresholds of FNN soft-sensor model and verify the established FNN soft-sensor model with the testing data.

## 4. Simulation Results

In this paper, 600 pieces of input data are selected as input and the concentrate grade and recovery rate are output of FNN soft-sensor model for the flotation process, where 550 samples are training data and the remaining 50 samples are testing data. Finally, the weights and thresholds of FNN are optimized by PSO-GSA algorithm. This paper selects the following five performance indexes to verify the prediction accuracy of different soft-sensor models, whose calculation equations are described as follows:

(18) NRMSE=1Tyd2∑t=1Tyt−ydt2,MSE=1T∑t=1Tyt−ydt2,MAPE=100T∑t=1Tyt−ydtydt,RMSE=1T∑t=1nydt−yt21/2,SSE=∑t=1nydt−yt2,

where *T* is the number of the prediction samples, *y*(*t*) is the predicted value, and *y*_*d*_(*t*) is the actual value.

**Figure 6:**
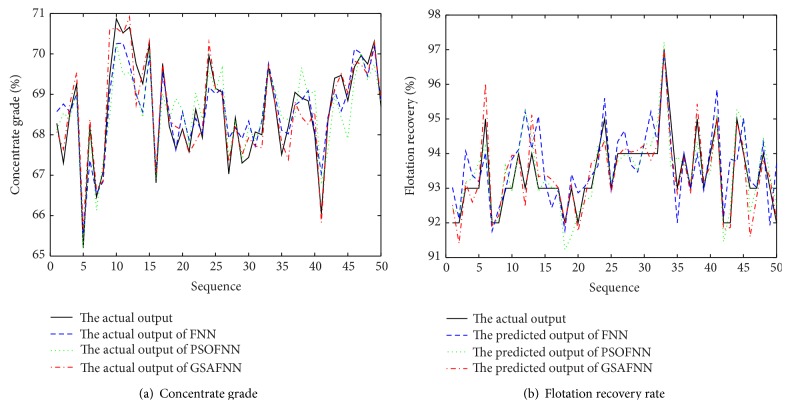
Predicted results of soft-sensor models.

**Figure 7:**
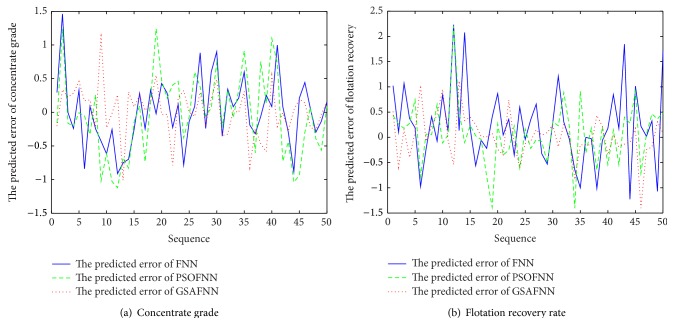
Predicted errors of soft-sensor models.

**Figure 8:**
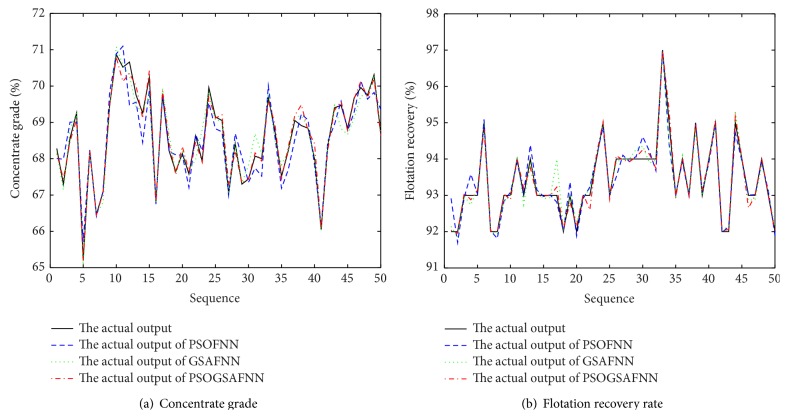
Predicted results of soft-sensor models.

**Figure 9:**
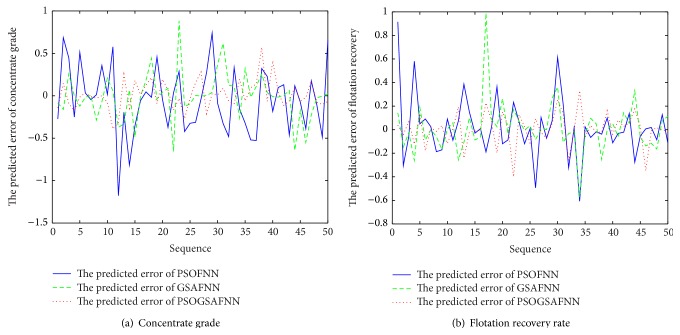
The predicted error of soft-sensor model.

The input dimension of FNN is 5, the number of neurons is 20 in the hidden layer, and the output dimension is 2. The activation function of FNN is tanh⁡ and the output uses the linear activation function. The initialization parameters of PSO-GSA algorithm are described as follows: *N* = 30, *c*_1_ = *c*_2_ = 2, *G*_0_ = 1, inertia weight *w* = 2, and the number of maximum iterations is *t* = 300. Firstly, three soft-sensor models based on FNN, FNN optimized by PSO algorithm, and FNN optimized by GSA are established to realize the prediction of concentrate grade and recovery rate in the flotation process.  Figure 6 is a comparison of the predicted output and the actual output under three models.  Figure 7 is a comparison of the output prediction error curves under three models. It can be seen, from the predicted output curves and the prediction error curves, in these three models, that FNN soft-sensor models optimized by PSO algorithm and GSA have better predictive accuracy than the standard FNN soft-sensor model. Therefore, in order to verify the validity of PSO-GSA hybrid algorithm, the proposed PSO-GSA FNN soft-sensor model is compared with PSO-FNN model and GSA-FNN model.  Figure 8 is a comparison of the predicted output and the actual output under three models.  Figure 9 is a comparison of the output prediction error curves under three models.

In order to compare the predictive ability and precision of these soft-sensor models based on the above-defined performance index, the performances are calculated and the results are shown in Table 2. Seen from Section 4, the prediction error of FNN soft-sensor model based on PSO-GSA is minimum.

## 5. Conclusion

The five variables (feed grade, feed concentration, feed flow, agents flow, and feed granularity) are selected as the input variables of the discussed soft-sensor model. The flotation concentrate grade and recovery rate are output variables. The hybrid algorithm combining PSO algorithm and GSA is used to optimize the parameters of FNN soft-sensor model in order to improve the predictive accuracy. It can be seen, from the prediction results and comparison results, that the FNN soft-sensor model based on the proposed PSO-GSA algorithm has the highest prediction accuracy compared with the other soft-sensor models, which can meet the online soft-sensor requirements of the real-time control in the flotation process.

## Figures and Tables

**Table 1:** Soft-sensor modeling data of flotation process.

Number	Auxiliary variables					Dominant variables	
	Feed concentration (%)	Feed flow (m^3^/h)	Feed grade (%)	Feed granularity (%)	Medicament flow (L/min)	Concentrate grade (%)	Recovery rate (%)
1	62.76	329	35	90	15.5	70.51	97.7
2	63.67	297	35	90	11.5	69.74	97.2
3	65.07	285	37	92	11.3	69.69	97.0
4	65.48	214	36	95	7.5	68.98	93.5
⋮	⋮	⋮	⋮	⋮	⋮	⋮	⋮
600	65.9	310	36	96	5.5	67.29	90.2

**Table 2:** Predictive performance comparison of soft-sensor models.

Predictive object	Predictive method	MSE	RMSE	NRMSE	SSE	MAPE
Concentrate grade (%)	FNN	0.2416	0.4915	0.0223	12.081	0.5564
	PSO-FNN	0.1528	0.3909	0.0008	7.6402	0.4471
	GSA-FNN	0.0764	0.2764	0.0006	3.8209	0.2649
	PSOGSA-FNN	0.0300	0.1733	0.0004	1.5009	0.1890

Flotation recovery rate (%)	FNN	0.1751	0.4184	0.0163	8.7535	0.3089
	PSO-FNN	0.0627	0.2505	0.0004	3.1372	0.1751
	GSA-FNN	0.0447	0.2114	0.0003	2.2341	0.1422
	PSOGSA-FNN	0.0194	0.1393	0.0002	0.9707	0.1054
